# Health Tract, No. 34

**Published:** 1862-02

**Authors:** 


### HEALTH TRACT, No. 34.

### HOW TO EAT.

Before a man becomes hungry, watchful nature has calculated, in her way, how much nutriment the body needs, and provides as much of a liquid substance as will be necessary to prepare from the food which may be eaten, that amount of sustenance which the system may require. When this is stored up, and all is ready, the sensation of hunger commences, and increases with the steadily increasing amount of the digesting material just referred to, and the very instant the first mouthful of food is swallowed, this "gastric juice" is poured out into the stomach through a thousand sluices; but no more has been prepared than was necessary, for Nature does nothing in vain; so that if a single mouthful more of food has been swallowed than the untempted or unstimulated appetite would have called for, there is no gastric juice for its solution, and it remains but to fret and worry and irritate for hours together. If the amount eaten is much in excess, the stomach, as if in utter discouragement at the magnitude of its task, ceases its attempts at digestion, and forthwith commences the process of ejecting the unnatural load by means of nausea and vomiting in some cases; in others, it remains for an hour or more like a weight, a hard round ball, or a lump of lead, an uneasy heaviness; then it begins to "sour," that is, to decompose, to rot, and the disgusting gas or liquid comes up into the throat, causing more or less of a scalding sensation from the pit of the stomach to the throat; this is called "heartburn." At length, the half-rotted mixture is forced out of the mouth by the outraged stomach with that horrible odor and taste with which every glutton is familiar. In some cases the stenchy mass is passed out of the stomach downwards, causing, in its progress, a gush of liquid from all parts of the intestinal canal, to wash it, with a flood, out of the system; this is the "Diarrhea" which surprises the gourmand at midnight or in the early morning hours, when a late or over-hearty meal has been eaten. When sufficient food has been taken for the amount of gastric juice supplied, hunger ceases, and every mouthful swallowed after that, no gastric juice having been prepared for its dissolution, remains without any healthful change, inflaming, and irritating, and exhausting the stomach by its efforts to get rid of it, and this is the first step towards forming "dyspepsia," which becomes more and more deeply fixed by every repeated outrage, until at length it remains a life-time worry to the mind, filling it with horrible imaginings, and a wearing wasting torture to the body, until it passes into the grave.

The moral of the article is, that the man who "forces" his food, he who eats without an inclination, and he who strives by tonics, or bitters, or wine, or other alcoholic liquors, to "get up" an appetite, is a sinner against body and soul—a virtual suicide!

